# On the Application of Carbolic Acid as a Local Anæsthetic in Surgical Operations

**Published:** 1871-09

**Authors:** J. H. Bill

**Affiliations:** Surgeon U. S. Army


### On the Application of Carbolic Acid as a Local Anæsthetic in Surgical Operations.

By Dr. J. H. Bill, Surgeon U. S. Army.

In conducting some investigations on the action, &c., of carbolic acid, the writer has made the following observations, which, as they seem to be of practical importance, he communicates in advance of the other results:—

All who have handled this substance must have noticed the tingling sensation, not unlike that produced by aconite, in the finger tips and other parts touched by the acid, which presently passes in a greater or less anæsthesia. On trying to determine the amount of this anæsthesia with an ordinary æsthesiometer it was found not only impossible to distinguish two points, however widely separated, but even to recognise the presence of one.

The prick of the point was not felt at all as pain, nor was an incision productive of uneasiness. The experiment was, therefore, extended thus :—The radial side of the writer's left forearm was covered with a cloth soaked in a saturated solution for a half hour, then a streak was traced over the course of the radial artery with a camel's hair brush dipped in acid liquefied by one-twentieth bulk of water. This streak extended from the styloid process to near the elbow, and after a few minutes was rubbed off. An incision was then made with a common scalpel from a point about two inches above the styloid process towards the internal condyle for five inches, occupying as near as possible the middle of the streak made with the brush, and extending down to the fasciæ investing the flexor muscles [superficial] of the thumb and fingers, so that at its lower extremity the radial artery was exposed and could have been ligated. This incision was unattended with pain, save where nerves distributed to or passing over the muscular fasciæ were pricked or divided, and even in this case the pain was not at all unbearable. *The incision af the integument was painless*, and the writer would have been unconscious of the injury save from the sensation communicated to his hand holding the knife as it was drawn through the tissues This observation or experiment was made nearly a year ago. It was applied practically at once to all minor cutting operations. The writer has not incised a felon or bubo since without successfully employing this method for preventing or greatly mitigating pain. Many cases could be given, one will suffice.—David Harris, of Vancouver, applied with his second finger of the left hand highly inflamed from a felon, the parts much injured by burrowing of pus. A previous felon had been treated on another finger a few months before, and the requisite incision had given him exqusite pain ; the patient therefore apprehended great suffering from any operation on the finger now diseased, and begged for chloroform. However, the finger was soaked for fifteen minutes in warm water containing three per cent. of carbolic acid, dried, and then the brush dipped in the concentrated acid, drawn over the finger in the course of the intended incision. These, two in number, were then made, using a thin edged scalpel by a slow sawing motion, allowing only the weight of the knife to make the cut. The patient stated that he had suffered no pain, or not more than would have resulted from handling the parts. The parts healed at once. Sometimes it is necessary after making an incision nearly through the integument, if sensibility becomes apparent, to brush out the wound made with some liquefied acid before extending the incision deeper. This was necessary in a palmar abscess treated by this method without pain. The writer has excised a small tumour partly by this plan. Buboes have been operated on painlessly, and in short, the writer can recommend the plan in any cutting operation where no dissection of the skin is involved, and where all the pain results from the cutting of the skin. It is hoped that it will be of special service to those who are compelled to operate without an assistant. The writer was thus compelled this summer to remove from the right hand, by an incision of over two inches in length, a large wooden splinter which had been thrust through the palmar fascia, and had lodged under the tendon of the lumbricalis of the index finger. It was done without pain, save where a nerve was divided. The incision healed without a scar.

These facts, which the writer believes he is the first to point out, have theoretical relations of great interest which may be discussed in a future paper.—*American Jour. of Med. Sciences, Oct.* 1870, *p.* 573.—*Braithwaite's Retrospect.*

